# Regulation of IL-22BP in psoriasis

**DOI:** 10.1038/s41598-018-23510-3

**Published:** 2018-03-23

**Authors:** Stefanos Voglis, Sonja Moos, Luise Kloos, Florian Wanke, Morad Zayoud, Penelope Pelczar, Anastasios D. Giannou, Silvia Pezer, Michael Albers, Felix Luessi, Samuel Huber, Knut Schäkel, Florian C. Kurschus

**Affiliations:** 1 Institute for Molecular Medicine, University Medical Center of the Johannes Gutenberg-University Mainz, Mainz, Germany; 2 I. Medizinische Klinik und Poliklinik, Universitätsklinikum Hamburg-Eppendorf, 20246 Hamburg, Germany; 3 Department of Dermatology, Heidelberg University Hospital, 69120 Heidelberg, Germany; 4 Department of Research, Phenex Pharmaceuticals AG, Heidelberg, Germany; 5 Department of Neurology, University Medical Center of the Johannes Gutenberg University, Mainz, Germany; 6 Present Address: Department of Dermatology, Heidelberg University Hospital, Im Neuenheimer Feld 440, 69120 Heidelberg, Germany

## Abstract

IL-22 is a potent pro-inflammatory cytokine upregulated in psoriasis and in other inflammatory diseases. The function of IL-22 is regulated by the soluble scavenging receptor, IL-22 binding protein (IL-22BP or IL-22RA2). However, the role and regulation of IL-22BP itself in the pathogenesis of inflammatory disease remain unclear. We used the TLR7 agonist Imiquimod (IMQ) to induce a psoriasis-like skin disease in mice and found a strong downregulation of IL-22BP in the affected skin as well as in the lymph nodes of animals treated with IMQ. We also analysed psoriatic skin of patients and compared this to skin of healthy donors. Interestingly, IL-22BP expression was similarly downregulated in skin biopsies of psoriasis patients compared to the skin of healthy donors. Since IL-22BP is expressed foremost in dendritic cells, we characterized its expression in monocyte-derived dendritic cells (MoDC) during maturation. In this way, we found Prostaglandin E2 (PGE_2_) to be a potent suppressor of IL-22BP expression *in vitro*. We conclude that regulation of IL-22BP by inflammatory mediators is an important step for the progression of inflammation in the skin and possibly also in other autoimmune diseases.

## Introduction

IL-22 is a cytokine of the IL-10 family^1^ and signals by binding to the transmembrane IL-22 receptor (IL-22R), which consists of the two subunits IL-22R1 and IL-10R2^2^. Whereas expression of the IL-10R2 was found to be ubiquitous, the IL-22 specific IL-22R1 subunit is expressed primarily by tissue cells of non-hematopoietic origin such as epithelial and endothelial cells. Some reports also show expression of IL-22R1 by activated macrophages^3,4^. The major role of IL-22 is to initiate the innate tissue response to bacterial infections and to help tissue repair after infection or inflammation^5^. Through activation of the STAT3 signalling cascades, IL-22 induces proliferative and anti-apoptotic pathways that help to prevent tissue damage^5^. On the other hand, the cytokine has also been shown to play a crucial role in the pathogenesis of several autoimmune diseases^6^. Apart from the membrane bound receptor (IL-22R) of IL-22, the soluble scavenging receptor named *IL-22 binding protein* (IL-22BP or IL-22RA2)^7–9^ binds IL-22 with a considerable higher affinity (up to 2000 fold) compared to IL-22R^10,11^, thereby effectively inhibiting its biological activity.

Three different isoforms of IL-22BP, formed by alternative splicing, exist in humans^7,8,12^. In mice and rats, only one isoform exists, which corresponds to the human isoform 2 and has been described as the only isoform to efficiently bind and deactivate IL-22^12^. IL-22BP has been shown to be expressed by distinct dendritic cell populations in mesenteric lymph nodes and the gut of rodents^13,14^. It has also been recently shown that IL-22BP expressed by T cells plays an important role for IBD development in humans and IBD mouse models^15^. Furthermore, IL-22BP expression by human eosinophils in the gut may paly a detrimental role by inhibiting the protective effects of IL-22 in intestinal inflammation^16^. By blocking the effect of IL-22 not only *in vitro*^7–9^ but also *in vivo*^17^, IL-22BP has gained attention as a potential clinical target in IL-22 driven diseases. Recently, it has been shown that activation of the inflammasome with subsequent IL-18 production leads to down-regulation of IL-22BP expression by DC^14^.

Psoriasis vulgaris, a chronic autoimmune disease, is characterized by the infiltration of immune cells (e.g. mononuclear leukocytes and neutrophils) into the skin, as well as abnormal proliferation of keratinocytes, resulting in erythematous, scaly plaques in predilection sites^18^. A commonly used mouse model for psoriasis is induced by Aldara^19^, a 5% Imiquimod (IMQ)-containing crème usually prescribed for the treatment of hyperkeratotic skin disorders in humans. IMQ is a toll-like receptor 7 (TLR7) agonist, and therefore acts as an immune activator^20^. Topically applied to the mouse skin, it leads to a skin phenotype comparable to human psoriasis^21^. The IL-23/IL-17 axis is important in both human psoriasis and the IMQ-model^21,22^ and mice overexpressing IL-17A specifically in keratinocytes develop symptoms with high similarity to psoriasis in humans^23^. The Th17-derived IL-22 has been shown to play a critical role in IL-23 induced acanthosis in mouse skin^24^ and in the development of psoriasis-like disease models^25^, particularly in the IMQ-induced psoriasis mouse model^26^. Furthermore, elevated IL-22 mRNA and protein levels in lesional skin and blood of psoriasis patients have been described^27,28^.

We investigated the expression and regulation of IL-22BP in human monocyte-derived dendritic cells (MoDC) as well as in skin from IMQ-treated mice and psoriasis patients. We found a strong regulation of IL-22BP expression in MoDC by prostaglandin E2 (PGE_2_). Furthermore, we showed that IL-22BP expression is strongly downregulated in psoriatic skin both from mice and humans and in skin-draining lymph nodes of mice treated with Aldara crème.

## Results

### IL-22BP expression is downregulated in the skin and in the skin-draining lymph nodes (sdLN) of IMQ-induced psoriasis mice

**Figure 1:**
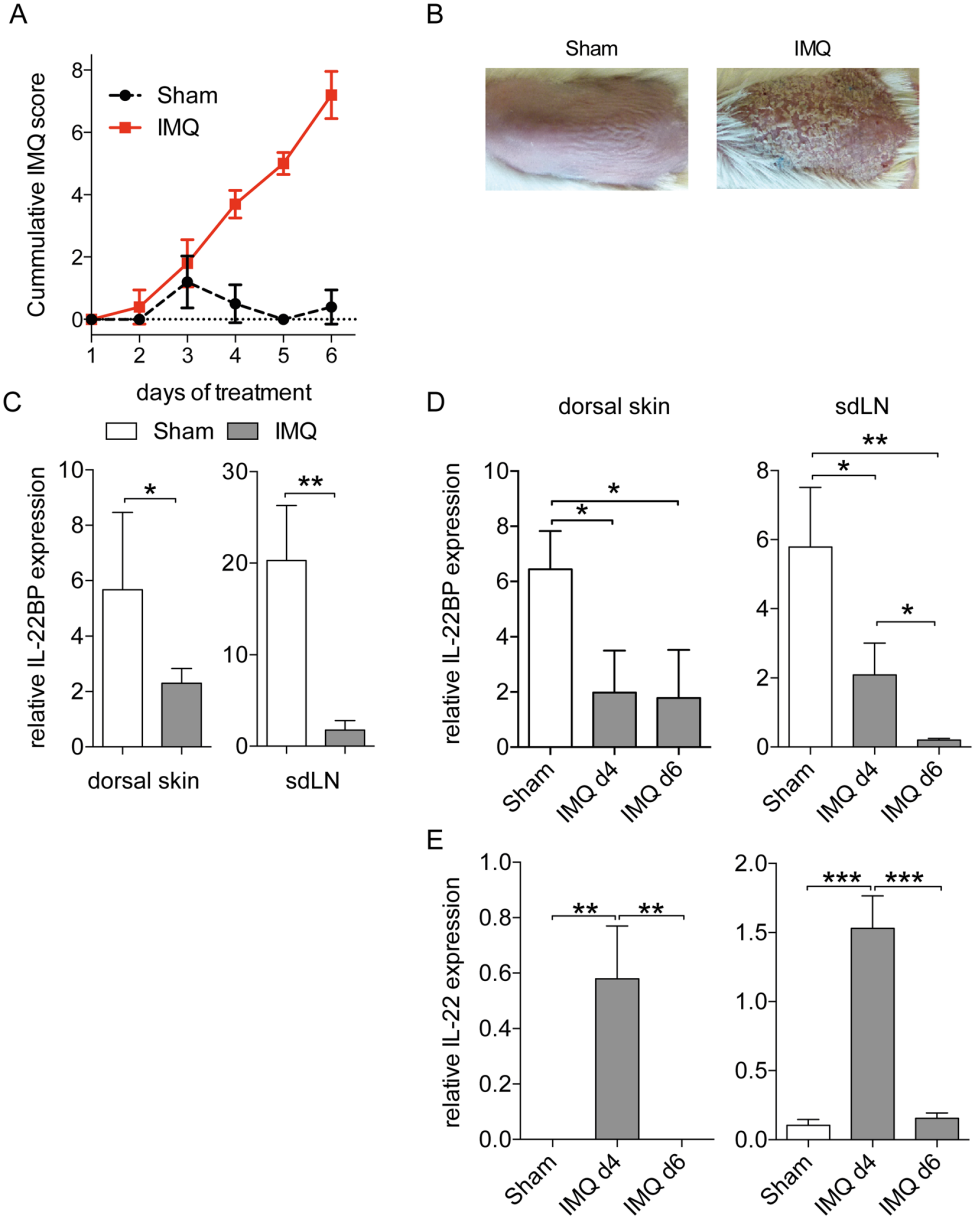
Downregulated IL-22BP expression in IMQ-induced psoriasis mice. (**A**) Dorsal skin thickness, erythema and scaling were scored daily and cleared to a modified PASI score. (IMQ and Sham treated mice each n = 5). (**B**) Representative dorsal skin of Sham- and IMQ-treated mice on day 6. (**C**) Quantitative RT-PCR from homogenates of dorsal skin and sdLN for the IL-22BP gene in IMQ and Sham-treated mice. Expression levels are shown relative to the housekeeping gene HPRT. (**D**,**E**) Quantitative RT-PCR from homogenates of dorsal skin and sdLN for the IL-22BP (**D**) and IL-22 (**E**) gene in IMQ-treated mice on day 4 and day 6 of disease course. For comparison the expression is shown for Sham treated mice, too. Expression levels are shown relative to the housekeeping gene HPRT.

IL-22 plays an important role for the development of IMQ-mediated psoriasis-like disease and probably also psoriasis^26,27^. We therefore hypothesized that IL-22BP might be involved in its regulation during the course of disease. Firstly, we used the IMQ-induced psoriasis-like mouse model^21^ and determined IL-22BP expression levels in different organs of the IMQ treated mice and controls (Sham-treatment)^29^. IMQ mice showed a clear dermal inflammation, exhibited by the thickening of dorsal skin and ears, as well as erythema (shown as cumulative IMQ score) (Fig. 1A) and plaque formation (Fig. 1B).

We found substantial IL-22BP mRNA expression in the dorsal skin as well as in the skin-draining lymph nodes (sdLN) of healthy mice (Fig. 1C). Furthermore, we detected a significant downregulation of its expression in the dorsal skin of the IMQ-treated mice. This finding was even stronger in the sdLN of the diseased animals. To better define the expression of IL-22BP and IL-22 we then analysed their expression in the various organs on different days (day 4 and 6) of disease development. In the dorsal skin of psoriatic mice, IL-22BP was already sufficiently downregulated on day 4, and there was no further decrease of its expression until day 6 (Fig. 1D). Yet in sdLN, the expression levels decreased to a level on day 6 on which almost no expression was detectable. In comparison, IL-22 showed a strong upregulation in its expression on day 4 in both dorsal skin and sdLN (Fig. 1E). Taking these findings together, we were able to show that IL-22BP is downregulated during disease development, whereas IL-22 is analogously upregulated. However, IL-22BP kept being downregulated from day 4 until the end of disease, whilst IL-22 expression was only abundant on day 4, and then either returned to normal levels (sdLN) or was undetectable (dorsal skin) after that.

### IL-22BP is also downregulated in human psoriatic skin

**Figure 2:**
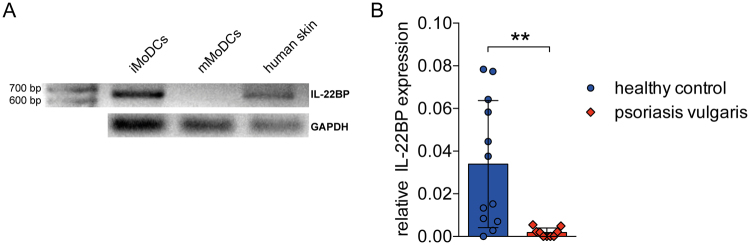
IL-22BP expression in human skin, and its expression pattern in lesional skin of psoriasis patients. (**A**) RT-PCR and subsequent gel electrophoresis from whole IL-22BP transcript of human skin. iMoDC as positive- mMoDC as negative-controls. GAPDH as internal control. (**B**) Quantitative RT-PCR from homogenates of skin biopsies from healthy controls (n = 12) or patients with psoriasis vulgaris (n = 9). The IL-22BP expression levels are shown relative to the housekeeping gene GAPDH.

We aimed to assess if our findings in mice were applicable to humans as well. We analysed skin biopsies of healthy donors to find out whether IL-22BP is expressed in human skin at all. IL-22BP is expressed in immature DC but downregulated upon maturation of DC^13,14^. Therefore, we used human immature monocyte derived DC (i)MoDC as a positive- and mature (m)MoDC as a negative-control for RT-PCR and found IL-22BP to be expressed in healthy human skin (Fig. 2A). Similarly to the IMQ-psoriasis mouse model, expression levels in biopsies of psoriasis vulgaris patients showed a strong downregulation of IL-22BP in the diseased skin compared to healthy controls (Fig. 2B).

### Regulation of IL-22BP in MoDC by PGE_2_

**Figure 3:**
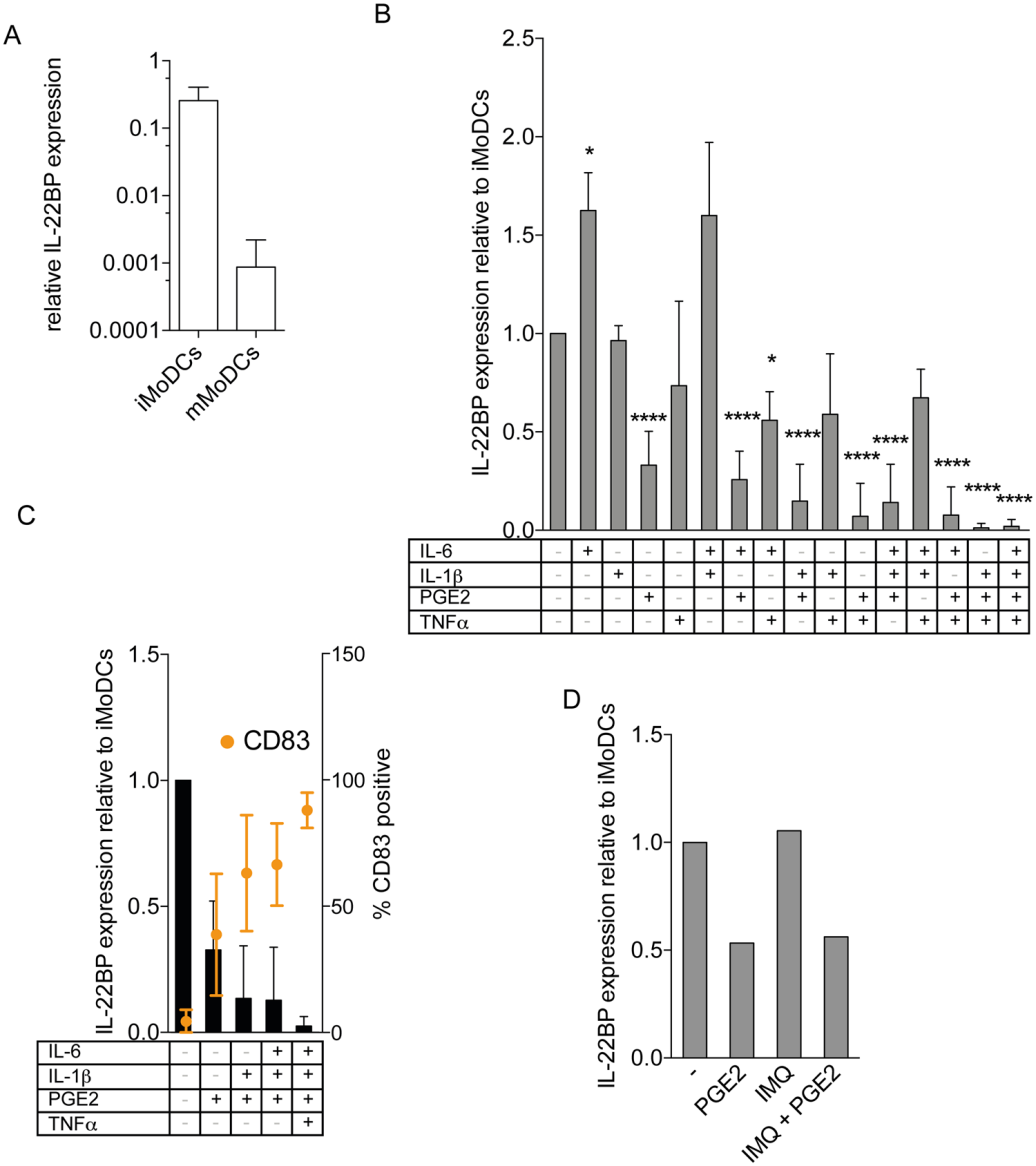
Regulation of IL-22BP expression in human MoDC. Quantitative RT-PCR from monocyte-derived DC (MoDC). Immature (i)MoDC were stimulated with different maturation-inducing cytokine combinations or with all necessary cytokines to generate mature (m)MoDC. IL-22BP expression levels are shown relative to housekeeping gene GAPDH (A) or DIMT1 (**B**,**C**). (**A**) Maturation of iMoDC leads to downregulation of IL-22BP. (**B**,**C**) Different cytokine combinations provoke IL-22BP downregulation by inducing maturation of iMoDC. (**C**) Upregulation of the DC maturation marker CD83 correlates with decreased expression of IL-22BP in MoDC upon stimulation. Quantitative RT-PCR for IL-22BP is shown on the left axis using black bars. Flow cytometric analysis of the DC maturation marker CD83 in stimulated MoDC; CD83 expression shown in percentage of CD11c^+^ cells on the right Y-axis with orange circles. (**D**) IMQ does not influence IL-22BP expressed on MoDCs. iMoDCs were stimulated either with PGE_2_ or IMQ (100 ng/mL) or with both. No effect on IL-22BP mRNA expression by IMQ was observed.

In humans, IL-22BP was first shown to be expressed by iMoDC^14^, and its expression was shown to be reduced during their maturation^13^, as also confirmed in this study (Fig. 3A). To determine potential proinflammatory factors leading to IL-22BP downregulation, we cultured iMoDC with various cytokine combinations, which are described to effectively induce maturation in MoDC^30^ (Supplementary Figure 1A). Within all the cytokine combinations tested, those including PGE_2_ showed the most effective downregulation of IL-22BP mRNA (Fig. 3B) and, correspondingly, a pronounced upregulation of the DC maturation markers CD80 and CD83 (Fig. 3C and Supplementary Figure 1). Interestingly, we found a slight but significant upregulation of IL-22BP expression upon stimulation with IL-6 (Fig. 3B). Since IMQ treatment led to downregulation of IL-22BP in mouse skin, we also tested its effect on IL-22BP expression using human MoDCs *in vitro*. In contrast to PGE_2_ treatment, we did not find any effect of IMQ on IL-22BP expression in MoDCs. In addition, together with PGE_2_, IMQ did not exert a synergistic effect on IL-22BP expression.

### Topical application of the COX-inhibitor Diclofenac on mouse skin does not alter IL-22BP expression in the IMQ-psoriasis model

**Figure 4:**
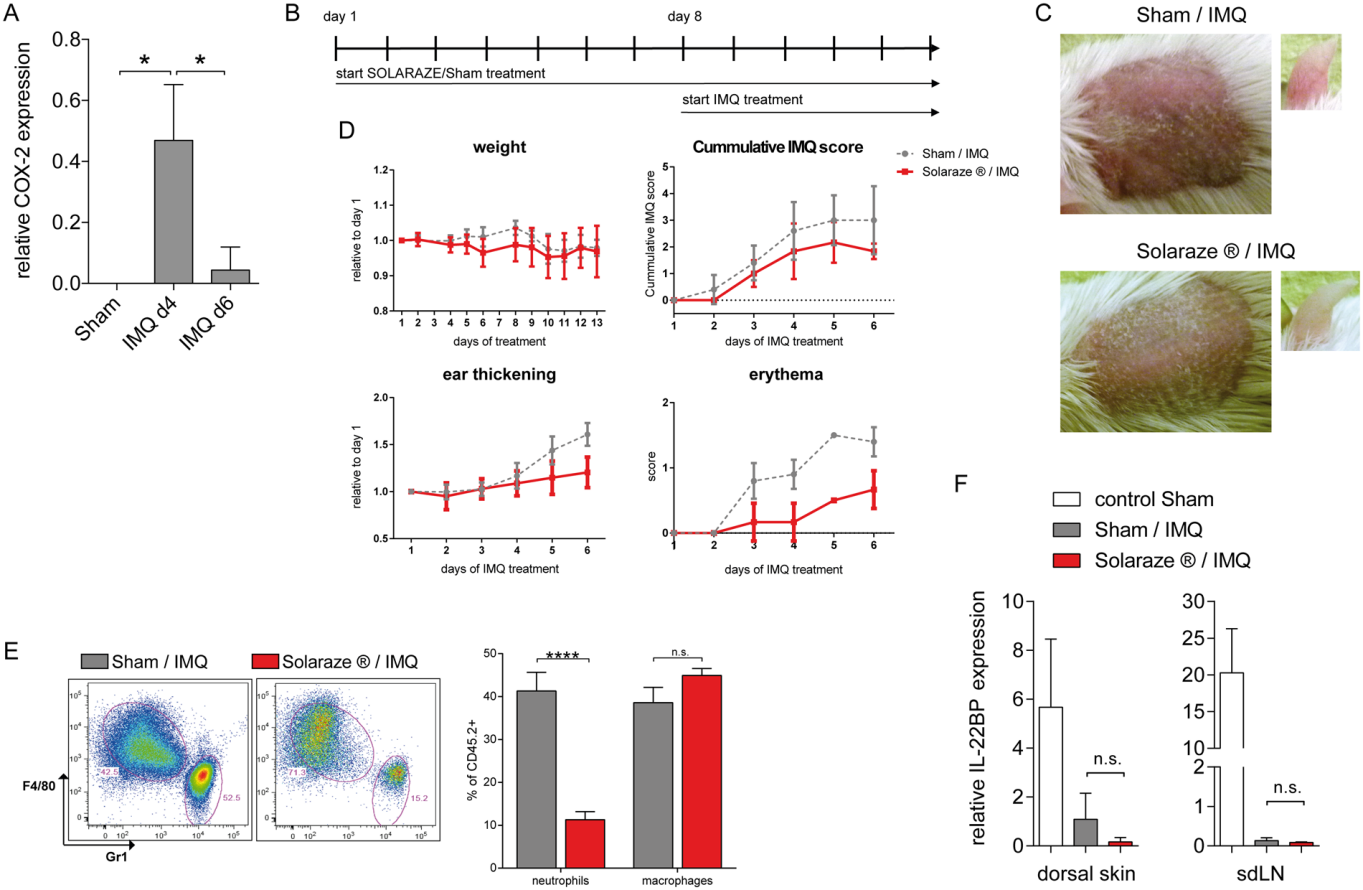
Inhibition of PGE_2_ synthesis with the NSAID Solaraze^®^ and its effect on IL-22BP expression. (**A**) Quantitative RT-PCR from homogenates of dorsal skin for the COX-2 gene in IMQ treated mice on day 4 and day 6 of the disease course. For comparison, the expression is also shown for Sham treated mice. Expression levels are shown relative to the housekeeping gene HPRT. (**B**) Treatment scheme: 7 subsequent days pre-treatment of the mice with either Solaraze^®^ or Sham gel. On day 8, additional parallel treatment with IMQ to induce the psoriasis-like skin disorder. (**C**) Representative dorsal skin and ears of Sham/IMQ and Solaraze^®^/IMQ treated mice on day 5 of IMQ treatment. (**D**) Dorsal skin thickness, erythema and scaling were scored daily and combined as a modified PASI score. In addition, change of weight and ear thickness (relative to day 1), as well as erythema (on a score from 0–4) are shown. (**E**) Flow cytometric analysis of single cell suspensions from digested ears of Sham/IMQ and Solaraze^®^/IMQ treated mice: Neutrophils (Gr-1^+^F4/80^−^) and macrophages (Gr-1^−^ F4/80^+^) are pre-gated on living CD19^−^, CD3ε^−^, CD45.2^+^, MHC-II^−^ and CD11b^+^ cells. Shown are representative plots. Bar graphs indicate neutrophil and macrophage infiltration in percentage to CD45.2^+^ cells. (**F**) Quantitative RT-PCR from homogenates of dorsal skin and sdLN for IL-22BP mRNA in Sham/IMQ and Solaraze^®^/Sham-treated mice. For comparison, the expression is also shown for Sham-treated mice. Expression levels are shown relative to the housekeeping gene HPRT.

Taking our findings in human MoDC into consideration, we hypothesized that the downregulation of IL-22BP in the IMQ-psoriasis mouse model might depend on PGE_2_ as well. We first analysed expression of Cyclooxygenase-2 (COX-2), one of the key rate-limiting enzymes in synthesis of PGE_2_^31^. The strongly elevated expression of COX-2 in the dorsal skin of IMQ-treated mice, with a peak on day 4 of disease course (Fig. 4A), seemed to sustain our hypothesis. We therefore attempted to verify whether the downregulation of IL-22BP in psoriatic mice depended on PGE_2_ by using the topical Diclofenac formulation Solaraze^®^– a *Nonsteroidal Anti-Inflammatory Drug* (NSAID) and therefore COX inhibitor^32^-containing gel. We pre-treated 7–9 week old mice for 7 consecutive days with either Solaraze^®^ or a Sham crème (containing the same ingredients except for the active agent Diclofenac). On day 8, we started the usual 5-day IMQ treatment protocol parallel to the Solaraze^®^/Sham treatment (Fig. 4B), with 6 hours in between the application of the 2 agents. Compared to the control group, the Solaraze^®^ treated mice showed reduced erythema (Fig. 4C,D) as well as less ear thickening (Fig. 4D). Furthermore, through FACS analysis of the ears, we detected a strongly decreased infiltration of neutrophils in the Solaraze^®^ group, while infiltration of macrophages showed no significant difference (Fig. 4E). Taken together, treatment with the COX inhibitor Solaraze^®^ partially ameliorated psoriasis-like symptoms. We analysed IL-22BP expression in dorsal skin and sdLN, but despite the effect of COX inhibition on IMQ mediated inflammation we did not find a significant difference between the two groups (Fig. 4F). Although Diclofenac showed a significant effect on development of IMQ-induced psoriasis-like disease with concomitant reduction of neutrophil infiltration, our results indicated that at least for the analysed time-point, PGE_2_ may not play a critical role in downregulation of IL-22BP in the mouse model.

## Discussion

We investigated the regulation of the soluble IL-22 scavenging receptor IL-22BP in humans and mice under inflammatory conditions *in vitro* and *in vivo*. We found IL-22BP mRNA to be strongly downregulated in MoDC upon PGE_2_ induced maturation, as well as in the inflamed mouse and human skin from psoriatic mice or from psoriasis patients respectively.

Regulation of the IL-22-scavenging protein in DC under pro-inflammatory conditions has been previously described as being mediated by maturation factors^13^ or by IL-18 secreted upon inflammasome activation^14^. In contrast to these findings, in our experimental settings, IL-18 treatment of MoDC did not reduce IL-22BP expression (not shown). Downregulation of IL-22BP is most likely necessary to allow IL-22 to function in the case of infections or tissue damage. Since IL-22 does also play a harmful role as in the case of psoriasis, one might anticipate intervention of IL-22BP downregulation as a potential treatment option for psoriasis patients. In contrast to our expectations, COX inhibition did not prevent downregulation of IL-22BP in the IMQ-psoriasis mouse model. In this case, perhaps alternative pro-inflammatory mediators such as cytokines might be responsible for the observed downregulation. Nevertheless, Diclofenac treatment of IMQ mice had a strong ameliorating effect on the disease course, with a much lower infiltration rate of neutrophils into the skin of these animals. Diclofenac, which was part of the solaraze crème used here, inhibits both COX-1 and COX-2 with a preference of COX-2. In this way it inhibits cyclic oxydation of arachidonat to PGH2, which is a precursor of multiple prostaglandine forms. Furthermore, Diclophenac may have effects on a multitude of other biochemical pathways^33^. Due to this widespread action, inhibition of PGE_2_ synthesis may only be one of many potential mechanisms by which Diclofenac ameliorates IMQ-mediated dermatitis. COX-inhibitor drugs are not commonly used in the treatment of psoriasis besides salicylic acid, which is used for removal of scales because of its keratolytic property.

It was shown in a very recent publication that IL-22BP deficient rats develop exacerbated IMQ-induced psoriatic-like lesions^34^. This is in line with our findings that IL-22BP needs to be downregulated for IL-22 in order to fulfil its role in the disease in patients as well as in mice. Although it is already downregulated by the inflammation, we consistently observed a residual expression of IL-22BP in skin. Therefore, deletion of IL-22BP allows IL-22 to act stronger under inflammatory settings in IL-22BP deficient animals than in WT animals. The same group also showed a downregulated level of IL-22BP mRNA in non-lesional skin of psoriasis patients compared to skin of healthy donors but an upregulation in lesional skin of these patients compared to non-lesional skin. Furthermore, they found upregulated levels of IL-22BP protein in the blood of psoriasis patients^34^. Since the report did not directly compare IL-22BP mRNA levels of healthy donors with that of diseased skin from psoriasis patients, our data cannot be directly compared. Our finding of a strong downregulation of IL-22BP mRNA in psoriatic skin compared to skin of healthy donors is in line with our mouse data and those of others that inflammatory cues down-modulate IL-22BP to enable IL-22 to act on local tissue cells.

In line with previous reports^13,14^, we found that it is also the case that in healthy mouse skin, dermal CD11b-positive DC but not lymphoid populations are the major IL-22BP expressing population (data not shown). Our initial screen of IL-22BP mRNA regulation was performed using published maturation cocktails for MoDC. In accordance with previous research^13^, we found that PGE_2_ was one of the main factors responsible for the observed downregulation, though the combination of all factors together as for mMoDC always showed a more complete downregulation than PGE_2_ alone. Furthermore, DC maturation was correlated with IL-22BP downregulation, indicating that PGE_2_ may indirectly contribute to this effect via its effect on DC maturation.

We show here, therefore, that IL-22BP is strongly regulated under inflammatory conditions in humans and in mouse models. Influencing this regulation may be an interesting possibility for the inhibition of IL-22’s harmful action in several autoimmune diseases.

## Material and Methods

### Mice

Experiments were performed using BALB/c mice purchased from either Harlan or Janvier. All mouse experiments were carried out in accordance with the relevant guidelines and regulations by the federal state Rhineland-Palatinate, Germany. Experiments were done after approval by the Landesuntersuchungsamt Rheinland-Pfalz with the individual animal experimentation application (TVA) # G13-1-099. The approval process contained an ethical committee meeting invited by the Landesuntersuchungsamt Rheinland-Pfalz.

### IMQ-induced psoriasis mouse model

7–9 week old female mice were shaved and had depilatory crème (Veet) applied to their backs. 2 days after hair removal, mice were treated either with Aldara (containing 5% IMQ; Meda AB) or Sham crème (homemade) on both ears (each 5 mg) and dorsal skin (50 mg) for 5 consecutive days^21^. The mice were sacrificed 24 h after the last treatment. To measure the severity of inflammation on the back, a scoring system considering skin thickness, scaling and erythema, (not taking into account the area which was determined by the experimenter) similar to the human PASI (Psoriasis Area and Severity Index) score was used (cumulative IMQ score)^29^.

### RNA isolation of mouse tissue and Quantitative Real-Time PCR

Dorsal skin and ears were homogenized and used directly for RNA extraction. Lymph nodes were first digested with DNase (10 μg/ml) (Roche) and Collagenase II (2 mg/ml) (Gibco) in phosphate-buffered saline (PBS). For RNA isolation, the peqGOLD Total RNA Kit (Peqlab) was used according to the manufacturer’s protocol. For reverse transcription, first strand cDNA was prepared by using the Superscript II kit (Invitrogen) with random primers, according to the manufacturers protocol. Quantitative real-time PCR (RT-PCR) was performed with the QuantiTect SYBR Green RT-PCR Kit (Qiagen) using the following primers: human IL-22BP (forward: 5′-GCC TGA ACA GTC ACA CTT GC-3′ reverse: 5′-GCG TTG ACT GAG TTC CTG CT-3′) whole human IL-22BP transcript (forward: 5′-GGC TTC CTC ATC AGT TTC TTC C-3′ reverse: 5′-TTC CAC ACA TCT CTC TTC ACT TCT C-3′), human GAPDH (forward: 5′-ATC GTG GAA GGA CTC ATG ACC A-3′ reverse 5′-CAG GGA TGA TGT TCT GGA GAG C-3′), human DIMT1 (forward 5′-GGC TGC CTT AAG ACC AAC TG-3′ reverse 5′-CGT GCC CTG AAC TCT TTT GT-3′) and mouse IL-22BP (forward: 5′-GAA GGT CCG ATT TCA GTC CA-3′ reverse: 5′-TCA CCC TCC CGT AAT ACA GC-3′). The following primers were purchased from Qiagen: mouse IL-22, mouse COX-2 and mouse HPRT. All changes in gene expression were calculated using the 2^−ΔΔCT^ method and are shown relative to the expression of the housekeeping genes hypoxanthine-guanine phosphoribosyltransferase (HPRT) in mice, and glyceraldehyde 3-phosphate dehydrogenase (GAPDH) or dimethyladenosine transferase 1 homolog (DIMT1) in human.

### Flow Cytometry

To prepare single-cell suspensions, ears were separated into dorsal and ventral parts and transferred in PBS. Digestion was performed with 0.05 mg/ml DNase I (Roche) and 0.04 mg/ml Liberase (Roche) in RPMI-1640 Medium (ThermoFisher Scientific) for 1 h at 37 °C. GentleMACS dissociator (Miltenyi Biotec) was used to homogenize the digested tissue. Single cell suspensions were treated with Fc-Block (BioXCell) and subsequently surface stained with the following monoclonal antibodies: MHC-II (BD Bioscience - 2G9), F4/80 (eBioscience - BM8), CD11b (eBioscience - M1/70), Gr1 (BD Bioscience - RB6-8C5), CD45.2 (eBioscience - 104), CD19 (Biolegend - 6D5), CD3ε (BD Bioscience - 145-2C11). Fixable Viability Dye eFluor450 (eBioscience) was used to exclude dead cells. All samples were acquired with FACS Canto II (BD Bioscience) and analysed with FlowJo (Treestar).

### Buffy coats and psoriasis-patient material

PBMCs from buffy coats of adult healthy donors (Center for Blood Transfusion, Mainz) were isolated by Ficoll-Hypaque gradient centrifugation. The subjects’ consent was obtained according to the Declaration of Helsinki (BMJ 1991; 302: 1194). Healthy and lesional psoriatic skin samples were collected under informed consent according to the Declaration of Helsinki (BMJ 1991; 302: 1194) and approved by the research ethics committee of the Medical Faculty of Heidelberg (S-306-2010). Psoriasis patients received at least 2–4 weeks no superficial treatment and had a mean PASI score of 13.9 ± 7.2. The samples, which had been stored immediately after collection at −80 °C, were thawed and homogenized with a TissueLyser (Qiagen). RNA extraction of the homogenates using peqGOLD TriFast (PeqLab) and reverse transcription using First Strand cDNA Synthesis Kit (Thermo Scientific) of the homogenized skin biopsies were performed according to the manufacturer’s protocol.

### Isolation of PBMCs from peripheral blood and MoDC generation

MoDC differentiation and maturation was performed in a similar way as to how has recently described^30,35^. PBMCs were isolated from blood by density gradient separation using Histopaque-1077 (Sigma-Aldrich). CD14^+^ cells were purified using anti-CD14 magnetic beads (Miltenyi Biotec) according to the manufacturer’s instruction. Purity was confirmed by flow cytometry and was above 95%. CD14^+^ cells were differentiated to immature (i)MoDC by stimulation with IL-4 (100 U/ml) and GM-CSF (400 U/ml) in X-Vivo 15 Medium (Lonza), supplemented with 2% autologous heat-inactivated plasma. The medium was changed on day 2 and 4. On day 6, different cytokine combinations were added for an additional 2 days to induce maturation of iMoDC. Cytokines were from PeproTech, Hamburg, Germany, GM-CSF (400 (d0), 800 (d2 and d4) U/ml), IL-4 (100 U/ml), IL-6 (1000 U/ml), IL-1β (10 ng/ml), TNF-α (10 ng/ml) and PGE_2_ (2 µM) or as indicated from Sigma-Aldrich, Germany.

### Statistical analysis

Graphs were made using GraphPad Prism. Statistical significance was calculated using the unpaired Student’s t-test. Values of p ≤ 0.001, p ≤ 0.01 and p ≤ 0.05 are displayed by three, two or one asterisks respectively. Data is represented as mean with SD if not stated otherwise.

### Data Availability

The datasets generated during and/or analysed during the current study are available from the corresponding author upon reasonable request.

## Electronic supplementary material

Supplementary Figure 1

## References

[1] Dumoutier L, Louahed J, Renauld JC (2000). Cloning and characterization of IL-10-related T cell-derived inducible factor (IL-TIF), a novel cytokine structurally related to IL-10 and inducible by IL-9. Journal of immunology.

[2] Bleicher L (2008). Crystal structure of the IL-22/IL-22R1 complex and its implications for the IL-22 signaling mechanism. FEBS Lett.

[3] Ke Y, Sun D, Jiang G, Kaplan HJ, Shao H (2011). IL-22-induced regulatory CD11b + APCs suppress experimental autoimmune uveitis. Journal of immunology.

[4] Dhiman R (2009). IL-22 produced by human NK cells inhibits growth of Mycobacterium tuberculosis by enhancing phagolysosomal fusion. Journal of immunology.

[5] Wolk K, Witte E, Witte K, Warszawska K, Sabat R (2010). Biology of interleukin-22. Semin Immunopathol.

[6] Nikoopour E, Bellemore SM, Singh B (2015). IL-22, cell regeneration and autoimmunity. Cytokine.

[7] Dumoutier L, Lejeune D, Colau D, Renauld JC (2001). Cloning and characterization of IL-22 binding protein, a natural antagonist of IL-10-related T cell-derived inducible factor/IL-22. J Immunol.

[8] Xu W (2001). A soluble class II cytokine receptor, IL-22RA2, is a naturally occurring IL-22 antagonist. Proc Natl Acad Sci USA.

[9] Kotenko SV (2001). Identification, cloning, and characterization of a novel soluble receptor that binds IL-22 and neutralizes its activity. J Immunol.

[10] Wolk K (2007). IL-22 induces lipopolysaccharide-binding protein in hepatocytes: a potential systemic role of IL-22 in Crohn’s disease. Journal of immunology.

[11] Jones BC, Logsdon NJ, Walter MR (2008). Structure of IL-22 bound to its high-affinity IL-22R1 chain. Structure.

[12] Weiss B (2004). Cloning of murine IL-22 receptor alpha 2 and comparison with its human counterpart. Genes Immun.

[13] Martin JC (2014). Interleukin-22 binding protein (IL-22BP) is constitutively expressed by a subset of conventional dendritic cells and is strongly induced by retinoic acid. Mucosal Immunol.

[14] Huber S (2012). IL-22BP is regulated by the inflammasome and modulates tumorigenesis in the intestine. Nature.

[15] Pelczar P (2016). A pathogenic role for T cell-derived IL-22BP in inflammatory bowel disease. Science.

[16] Martin JC (2016). IL-22BP is produced by eosinophils in human gut and blocks IL-22 protective actions during colitis. Mucosal Immunol.

[17] Sugimoto K (2008). IL-22 ameliorates intestinal inflammation in a mouse model of ulcerative colitis. J Clin Invest.

[18] Lowes MA, Bowcock AM, Krueger JG (2007). Pathogenesis and therapy of psoriasis. Nature.

[19] Kurschus F (2016). Of men and mice: analysing the action of an established drug using tumour necrosis factor-alpha-deficient mice in the imiquimod psoriasis model. Br J Dermatol.

[20] Gilliet M (2004). Psoriasis triggered by toll-like receptor 7 agonist imiquimod in the presence of dermal plasmacytoid dendritic cell precursors. Arch Dermatol.

[21] van der Fits L (2009). Imiquimod-induced psoriasis-like skin inflammation in mice is mediated via the IL-23/IL-17 axis. J Immunol.

[22] Di Cesare A, Di Meglio P, Nestle FO (2009). The IL-23/Th17 axis in the immunopathogenesis of psoriasis. J Invest Dermatol.

[23] Croxford AL (2014). IL-6 Regulates Neutrophil Microabscess Formation in IL-17A-Driven Psoriasiform Lesions. J Invest Dermatol.

[24] Zheng Y (2007). Interleukin-22, a T(H)17 cytokine, mediates IL-23-induced dermal inflammation and acanthosis. Nature.

[25] Ma HL (2008). IL-22 is required for Th17 cell-mediated pathology in a mouse model of psoriasis-like skin inflammation. The Journal of clinical investigation.

[26] Van Belle AB (2012). IL-22 is required for imiquimod-induced psoriasiform skin inflammation in mice. J Immunol.

[27] Wolk K (2006). IL-22 regulates the expression of genes responsible for antimicrobial defense, cellular differentiation, and mobility in keratinocytes: a potential role in psoriasis. European journal of immunology.

[28] Boniface K (2007). A role for T cell-derived interleukin 22 in psoriatic skin inflammation. Clin Exp Immunol.

[29] El Malki K (2013). An alternative pathway of imiquimod-induced psoriasis-like skin inflammation in the absence of interleukin-17 receptor a signaling. J Invest Dermatol.

[30] Jonuleit H (1997). Pro-inflammatory cytokines and prostaglandins induce maturation of potent immunostimulatory dendritic cells under fetal calf serum-free conditions. European journal of immunology.

[31] Rouzer CA, Marnett LJ (2009). Cyclooxygenases: structural and functional insights. Journal of lipid research.

[32] Simmons DL, Botting RM, Hla T (2004). Cyclooxygenase isozymes: the biology of prostaglandin synthesis and inhibition. Pharmacological reviews.

[33] Gan TJ (2010). Diclofenac: an update on its mechanism of action and safety profile. Current medical research and opinion.

[34] Martin JC (2017). Limited Presence of IL-22 Binding Protein, a Natural IL-22 Inhibitor, Strengthens Psoriatic Skin Inflammation. J Immunol.

[35] Zayoud M (2013). Subclinical CNS inflammation as response to a myelin antigen in humanized mice. J Neuroimmune Pharmacol.

