# Supplementary material for: Regulation of IL-22BP in psoriasis
Source: Sci Rep. 2018 Mar 23;8:5085. doi: 10.1038/s41598-018-23510-3 (PMC5865214; doi:10.1038/s41598-018-23510-3)
Supplement: Supplementary file 1 — Supplementary Figure 1 [file 41598_2018_23510_MOESM1_ESM.pdf]

Supplementary Information

Regulation of IL-22BP in psoriasis

Stefanos Voglis[1,2], Sonja Moos[1,2], Luise Kloos[1], Florian Wanke[1], Morad Zayoud[1], Penelope Pelczar[3], Anastasios D. Giannou[3], Silvia Pezer[4], Michael Albers[5], Felix Luessi[6], Samuel Huber[3], Knut Schäkel[4] and Florian C. Kurschus[1,7,8]

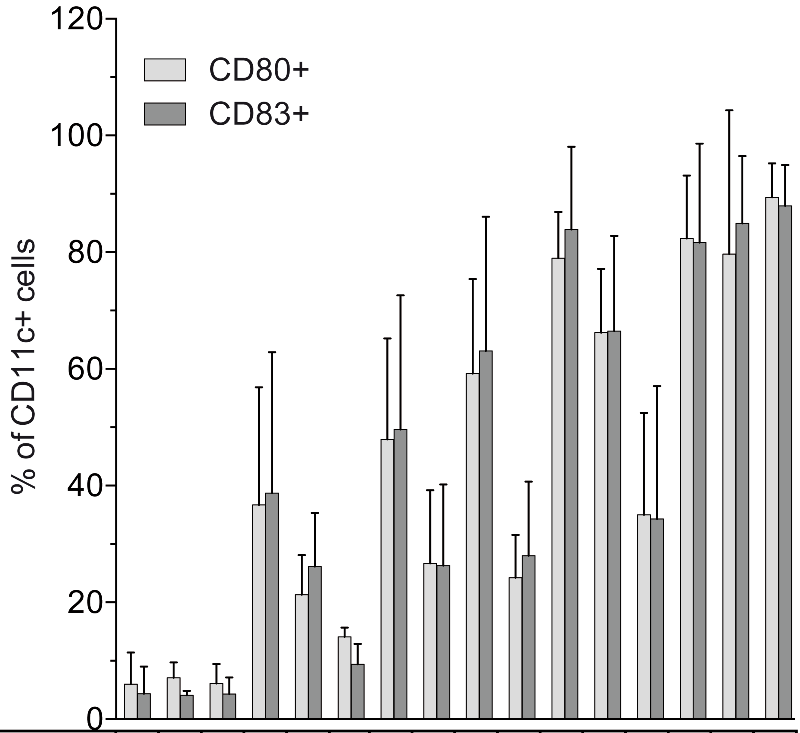

IL-6	-	+	-	-	-	+	+	+	-	-	-	+	+	+	-	+
IL-1β	-	-	+	-	-	+	-	-	+	+	-	+	+	-	+	+
PGE2	-	-	-	+	-	-	+	-	+	-	+	+	-	+	+	+
TNFα	-	-	-	-	+	-	-	+	-	+	+	-	+	+	+	+

Supplementary Figure 1: Flow cytometric analysis of DC maturation markers CD80 and CD83 in MoDC. Bar graphs indicate percentage of CD80 and CD83 expressing CD11c^{+} cells. iMoDC were stimulated with different maturation-inducing cytokine combinations or with all necessary cytokines to generate mMoDC.
